# Occult and Overt HBV Co-Infections Independently Predict Postoperative Prognosis in HCV-Associated Hepatocellular Carcinoma

**DOI:** 10.1371/journal.pone.0064891

**Published:** 2013-06-21

**Authors:** Ming-Ling Chang, Yu-Jr Lin, Chee-Jen Chang, Charisse Yeh, Tse-Ching Chen, Ta-Sen Yeh, Wei-Chen Lee, Chau-Ting Yeh

**Affiliations:** 1 Liver Research Center and Department of Hepato-Gastroenterology, Chang Gung Memorial Hospital, Linko, Taiwan; 2 Graduate Institute of Clinical Medical Sciences, Chang Gung University, College of Medicine, Linko, Taiwan; 3 Resource Center for Clinical Research, Chang Gung Memorial Hospital, Linko, Taiwan; 4 Flintridge Sacred Heart Academy, La Canada, Flintridge, California, United States of America; 5 Department of Pathology, Chang Gung Memorial Hospital, Taipei, Taiwan; 6 Division of General Surgery, Department of Surgery, Chang Gung Memorial Hospital, Taipei, Taiwan; Singapore Institute for Clinical Sciences, Singapore

## Abstract

**Objective and Background:**

The roles of chronic hepatitis B virus (HBV) co-infection (CI) in carcinogenesis of hepatitis C virus (HCV)-associated hepatocellular carcinoma (HCC) remained controversial. To gain new insights into this issue, we investigated the postoperative prognostic value of HBVCI in HCV-associated HCC.

**Methods:**

A study cohort of 115 liver tissues obtained from the noncancerous parts of surgically removed HCV-associated HCCs were subjected to virological analysis in a tertiary care setting. Assayed factors included clinicopathological variables, tissue amounts of viral genomes, genotypic characterization of viruses, as well as the presence of overt (serum HBsAg positive) or occult (serum HBsAg negative but tissue HBV-DNA positive) HBVCI. Cox proportional hazard model was used to estimate postoperative survivals.

**Results:**

Of the 115 patients, overt and occult HBVCIs were detected in 35 and 16 patients, respectively. Multivariate analysis revealed that tumor size >3 cm (adjusted hazard ratio (AHR), 2.079 [95% confidence interval, 1.149∼3.761]), alpha-fetoprotein >8 ng/mL (AHR, 5.976 [2.007∼17.794]) albumin <4 g/dL(AHR, 2.539 [1.399∼4.606]), ALT >50 U/L (AHR,1.086 [1.006∼1.172]), presence of occult HBVCI (AHR, 2.708 [1.317∼5.566]), and absence of overt HBVCI (AHR, 2.216 [1.15∼4.269]) were independently associated with unfavorable disease-free survival. Patients with occult HBVCI had a shorter disease-free (*P* = 0.002), a shorter overall survival (*P* = 0.026), a higher bilirubin level (*P* = 0.003) and a higher prevalence of precore G1896A mutation (*P* = 0.006) compared with those with overt HBVCI.

**Conclusion:**

Occult and overt HBVCI served as independent predictors for postoperative survival in HCV-associated HCC.

## Introduction

Hepatocellular carcinoma (HCC) accounts for >90% of primary liver malignancies and ranks the fifth most common solid cancer and the third leading cause of cancer-related death worldwide [1]. It is multifactorial in origin. About three-quarters of HCCs are attributed to chronic hepatitis B virus (HBV) and hepatitis C virus (HCV) infections [2]. Other risk factors include heavy alcohol drink, liver cirrhosis, old age, male gender, underlying chronic liver diseases, aflatoxin exposure and diabetes [3].

HCV infection is the most important risk factor for HCC in Hispanics and African Americans, whereas HBV infection is the dominant risk in Asians [4]. An increasing incidence of HCC has been reported in Japan and Western countries in recent years, mainly attributed to increased prevalence of HCV infection; while the contribution of HBV is declining [4], [5]. Similar trend has been observed in Taiwan, an area hyperendemic for HBV infection, where the seroprevalence of hepatitis B surface antigen (HBsAg) among patients with HCC was >90% three decades ago. Recently, however, chronic HCV infection was found in >30% of HCC patients and the prevalence of HBV decreased to about 70% [6]. On account of similar transmission modes between HBV and HCV, coinfection with the two viruses is not uncommon [7]. At least three distinct clinical states of HBV viral persistence have been defined based on serological findings: chronic hepatitis B, silent or “healthy” carrier, and occult hepatitis B [8]. Occult HBV infection is characterized by persistence of HBV DNA in the liver of individuals negative for serum HBsAg [9]. Although HCV co-infection leads to decrease of HBV replication and expression of HCV core protein in liver cells results in reduced expression of HBV viral proteins were ever shown [8], some *in vitro* experiments of co-transfection failed to demonstrate the direct interference between HBV and HCV in the same cell [10], [11]. The viral interference observed in coinfected patients is probably due to indirect mechanisms mediated by innate and/or adaptive host immune responses [10], [11]. Anyhow, a high prevalence of occult HBV co-infection (HBVCI) has been found in patients with chronic HCV infection [12]. For example, in Japan, a hyperendemic area for HCV infection, the prevalence of occult HBVCI in patients infected with HCV ranged from 37 to 95% [13] comparing with 0 to 20% in patients of a control population [14].

Co-infection of HCV and HBV could result in more severe liver damage and thus accelerate the deterioration of liver function as well as the oncogenic process [15]. However, because of viral interference, coinfection of the two viruses might also reduce the replication of one or both of the viruses and thus counteract each other's effect on liver damage [16]. Whether there is a synergistic effect between HCV and HBV on the occurrence of HCC remains debatable. For overt HBVCI in HCV infected patients, a meta-analysis of 32 studies concluded that the joint effects of HBV and HCV on the etiology of HCC was between additive and multiplicative [17]. However, another meta-analysis including 59 studies suggested that HBV and HCV co-infection for HCC risk was not significantly greater than HBV or HCV mono-infection [18]. As to occult HBVCI, several studies, mostly from Europe and Japan, have found a higher prevalence of occult HBVCI in patients with HCV-associated HCC when compared with HCV-infected patients with no HCC [8], [19]. However, a study from United States revealed that neither previous nor occult HBVCI constituted an important risk factor in HCC development [20].

Despite the accumulated reports on virological factors associated with the development of HCV-related HCC [21], studies are still lacking, regarding the prognostic value of the hepatic virological factors (including HBVCI) in HCV-associated HCC. Previously, we have demonstrated the feasibility of examining the prognostic value of virological factors assayed directly from liver tissues in HBV-associated HCCs [22]. Because no grossly detectable tumor remains after surgical resection, these patients form a clinically homogeneous group. As such, the time to recurrence (disease-free survival) or death (overall survival) in these patients reflects the growth behavior of their HCCs. The identified host or virological predictors for postoperative survivals are thus considered tightly associated with development of liver cancer. With increasing prevalence of HCV-associated HCC worldwide, identification of key predictors for postoperative prognosis becomes more and more important. This task is particularly challenging in an HBV hyperendemic area, where HBVCI occurs in a significant proportion of HCV-associated HCC patients and may serve as an important contributing factor for prediction of postoperative survivals. In this study, we aimed to explore this issue.

## Methods

### Patients

This study was conducted under the approval of the institutional review board of Chang Gung Medical Center (CGMC). To conduct this study, the clinical records of 342 HCC patients receiving total removal of liver tumors from July 1998 to Aug 2001 in CGMC, Taiwan were reviewed. The liver tissues of these patients were stored in the tissue bank of CGMC after informed consents were obtained. Of them, 115 HCC patients who were positive for antibody against HCV (anti-HCV) and negative for antibody against hepatitis delta virus were included. All samples were frozen to –70°C, immediately after surgical resection until use. The following clinicopathological data were reviewed: gender, age, presence of liver cirrhosis, alcohol usage, Edmondson's histologic grade, microvascular invasion, macrovascular invasion, presence of tumor capsule, number of tumors, largest tumor size, presence of ascites upon surgery, alpha-fetoprotein (AFP), albumin, bilirubin, prothrombin time, creatinine, aspartate aminotransferase (AST), alanine aminotransferase (ALT), date of surgical resection, date of tumor recurrence, and date of last follow-up or HCC related death. In our medical center, patients with main portal vein thrombosis were excluded from surgical management. Minor portal vein invasion discovered during or after surgery was categorized as macrovascular invasion.

**Figure 2:**
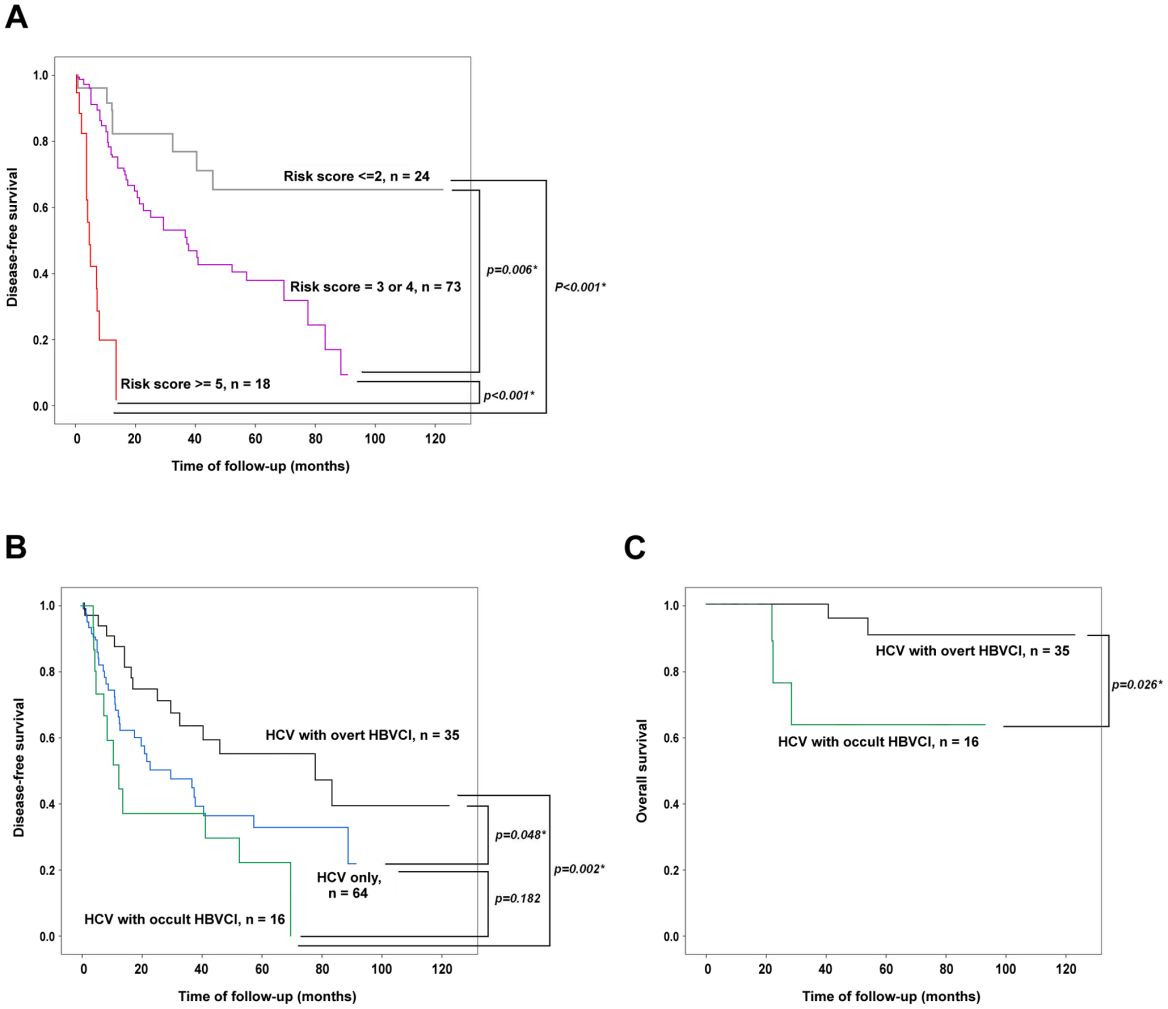
Kaplan-Meier analysis for HCV-associated HCC patients with various conditions of predictive factors. (A) Patients stratified by the risk score calculated from the 6 independent predictors. Grey line, risk score ≦2; Purple line, risk score 3 or 4; Red line, risk score ≥5. (B) Patients classified according to viral infection status. Black line, HCV infection with overt HBVCI; Blue line, HCV infection only; Green line, HCV infection with occult HBVCI. (C) Comparison of the overall survival between HCV-associated HCC patients with occult HBVCI (green line) and those with overt HBVCI (black line).

Similarly, the Cox proportional hazard model was used to examine the association between clinicopathological and virological factors and overall survival after surgical resection of HCV-associated HCC. No factor associated with overall survival was found in univariate analysis. However, patients with occult HBVCI had a significantly shorter overall survival compared with those with overt HBVCI (67.39±11.47 vs. 115.14±4.6 months, *P* = 0.026) (Figure 2C).

Preoperative diagnosis of HCC was made by one of the following methods: echo-guided liver biopsy, fine needle aspiration cytology, high AFP level (>200 ng/mL) plus at least one dynamic imaging study (dynamic computed tomography or magnetic resonance imaging), or one dynamic imaging study plus angiography (if AFP <200 ng/mL). Tumors were completely resected, with a safety-margin of >1 cm. Post-operative follow-up was performed by ultrasonography, chest X-ray, AFP, and blood biochemistry every 1 to 3 months in the first year and every 3 to 6 months thereafter. Abnormal findings were verified by computed tomography or magnetic resonance imaging. Intrahepatic recurrence was established by use of the aforementioned criteria. Extrahepatic recurrence was confirmed by biopsy, aspiration cytology, computed tomography or magnetic resonance imaging, with the choice of study dependent upon the location of the lesions as well as the condition of the patient.

HBsAg levels were measured by radioimmunoassay (Ausria-II, HBsAg-RIA; Abbott Laboratories, North Chicago, IL). Anti-HCV antibody levels were measured by a third-generation enzyme immunoassay (HCV EIA III; Abbott Laboratories). Antibodies against hepatitis delta antigen were detected by enzyme immunoassay (anti-HD; Formosa Biomedical Technology Corporation, Taiwan).

### Noncancerous hepatic and serum HCV RNA Quantification

Hepatic and serum HCV RNA was measured by COBAS TagMan HCV test (Roche Diagnostics, Tokyo, Japan). Serum levels were assayed according to the standard protocol provided by the manufacturer. For hepatic HCV-RNA assay, 20 mg of liver tissue was submitted for RNA extraction using the corresponding TRIzol reagents (Invitrogen, USA). The extracted samples were dissolved in pure water and submitted for the same standardized commercial assays. The final amounts of hepatic HCV RNA were calculated as IU per gram after corrected for the dilution factor, with a lower limit of detection of 1500 IU/g.

HCV genotype was determined by use of the InoLipa method (COBAS AmpliPrep/COBAS TaqMan HCV Test, Roche Diagnostics, Tokyo, Japan).

### Noncancerous hepatic and serum HBV DNA quantification

The hepatic and serum HBV-DNA concentration was quantified using Roche Tagman HBV Monitor (Roche Diagnostics, Basel, Switzerland). Serum levels were assayed according to the standard protocol provided by the manufacturer. For tissue HBV-DNA assay [23], 20 mg of liver tissue was submitted for DNA extraction using the corresponding TRIzol reagents. The extracted samples were dissolved in pure water and submitted for the same standardized commercial assays. The final amounts of hepatic HBV DNA were calculated as IU per gram after corrected for the dilution factor, with a lower limit of detection of 60 IU/g. In addition to the standardized assay, tissue HBV DNA was also detected by PCR using primers listed in Table 1.

**Table 1:** Nucleotide sequences of the primers used in this study.

Target regions	Stages of PCR	Primer sequences	Expected Sizes of PCR products
Pre-S			
	First	PSFo: 5′-ttgcgggtcaccttattcttg-3′	
		PSRo: 5′- agcaggggtcctaggagtc-3	598 bp
	Second	PSFi: 5′-ggaacaagagctacagc-3′	
		PSRi: 5′- ctgatgcgatgttctccatgtt-3′	558 bp
Precore/X			
	First	PCFo: 5′- gccttctcatctgccggtcc-3′	
		PCRo: 5′- gtatggtgaggtgaacaatg-3′	505 bp
	Second	PCFi: 5′- cataagaggactcttggactcc-3′	
		PCRi: 5′- aaagaattcagaaggcaaaaaaga-3′	320 bp
Virus-Host junction (Alu-PCR)			
	First	Alu3: 5′- aUUaacccUcacUaaagccUcgaUagaUYRYRccaYUgcac-3′	
		Alu5: 5′ – cagUgccaagUgUUUgcUgacgccaaagUgcUgggaUUa-3	
	Second	Tag3: 5′- attaaccctcactaaagcctcg-3′	
		Tag5: 5′- caagtgtttgctgacgccaaag-3′	

Y = c+t; R = a+g, U = dUTP; In Alu-PCR, four sets of PCR were performed using Alu3/PCFo, Alu3/PCRo, Alu5/PCFo, and Alu5/PCRo as the first stage PCR primers. Subsequently, uracil DNA glycosylase was added to break the DNA strands at apurinic dUTP sites. Finally, Tag3/PCFi, Tag3/PCRi, Tag5/PCFi, and Tag5/PCRi were used respectively for the second stage PCR.

### Polymerase chain reaction (PCR) for detection of intrahepatic HBV DNA

Nested PCRs targeted to the Pre-S and precore regions were performed by use of the intrahepatic HBV DNA as a template. The primer sequences and product sizes were listed in the Table 1. Because the precore and X genes were in fact overlapped, the primers used to detect precore region in table 1 in fact encompassed more than 1/3 of the X gene region. Amplified PCR products were analyzed by electrophoresis on an 1.0% agarose gel and transferred to a Hybond-N+ nylon membrane for Southern blot analysis to verify the specificity (Amersham-Pharmacia, Buckinghamshire, UK).

### Detection of HBV mutants

The methods to detect HBV basal core promoter (BCP) A1762T/G1764A mutations, precore stop codon G1896A mutation and pre-S deletion were performed as previously described [22]. If necessary, the whole HBV pre-S/S gene was sequenced to search for mutations at the “a” determinants [24].

### Detection of HBV DNA integration in noncancerous liver tissues

A PCR based technique (Alu-PCR) was employed using specific primers directed to human Alu sequences and to HBV sequences to amplify virus-host junctions, according to previous publications [25], [26]. Primer sets of this PCR strategy were listed in the Table 1. We modified this Alu-PCR method to use primers specifically directed to the integration sites located between DR1 and DR2 regions. Amplified PCR products were analyzed by Southern blot using a probe specific to HBV genome. To prepare a full length HBV probe, the total HBV genome was amplified and labeled with digoxigenin (Roche, Mannheim, Germany).

### Histopathology and immunohistochemistry analysis for HBV viral proteins

Immunohistochemistry for HBV antigens was performed on sections from paraffin-embedded liver biopsies by use of the Avidin Biotin Complex method. The primary antibodies used were the mouse monoclonal antibodies against HBsAg (Chemicon, Temecula, CA) and HBc Ag (Dako, Carpinteria, CA), respectively. Visualization was achieved through reaction of the substrate 3,3′-Diaminobenzidine. The samples from patients with negative serum HBsAg and negative tissue HBV DNA was taken as the negative controls while tissue samples from chronic hepatitis B patients as the positive controls. Intensity of staining was judged semiquantitatively as absent, minimal, moderate, or strong and staining pattern was reported as nuclear, cytoplasmic, sub-membranous, or absent, as described previously [27].

### Statistical analysis

Overall survival was calculated from the date of diagnosis to the date of death or last follow-up. Disease-free survival was measured from the date of diagnosis to the date of recurrence, metastasis, death or last follow-up. The Cox proportional hazard model was used to identify clinicopathological and virological factors associated with postoperative survivals. To determine the cutoffs of a factor with parametric data for Cox proportional hazard analysis, experimental univariate analysis was performed to evaluate the association between the factor and disease-free survival using a series of increasing values as the cutoffs. The experimental cutoffs were determined by using R version 2.13 (The R Foundation for Statistical Computing) which based on searching the highest coefficient after partition the variants from the classification and regression tree. Verifications of the experimental cutoffs were performed by subsequent stepwise Cox proportional hazard models, which were also used to predict independent predictors associated with disease-free survival. The Kaplan-Meier method was used to estimate the survival probability, and the log-rank test was used to compare the survival curves between groups. Statistical analysis to compare the variables among the patients with various virological statuses was performed by a one-way analysis of variance (ANOVA), and its significance was assessed by Sheffe's post-hoc test. Statistical analysis was conducted by the use of Statistical Product and Service Solutions (SPSS, version 17.0).

## Results

### Demographics and clinical features of the patients included

The demographics and clinical features of the patients with HCV-associated HCC were listed in Table 2. Of the 115 patients, 78 (67.8%) were males. Except for a higher prevalence of alcoholism in the male patients, no significant difference was observed between men and women with HCV-associated HCC in terms of cirrhosis, tumor number, largest tumor size, ascites, alpha-fetoprotein, albumin, bilirubin, prothrombin time, creatinine, aspartate aminotransferase (AST) and alanine aminotransferase (ALT). Of the enrolled patients, 35 (30.4%) had overt HBVCI with detectable serum HBsAg. In the remaining patients with undetectable serum HBsAg (totally 80 patients), 16 (20%) had detectable intrahepatic HBV DNA in the noncancerous liver tissue assayed by nested PCR targeted against two different HBV genomic regions.

**Table 2:** Basic clinical data of patients included.

Clinical parameters	Gender		P
	Female (n = 37)	Male (n = 78)	
Age (years)	64.3±9.4	62.2±11.7	0.333
Cirrhosis	27 (73.0%)	50 (64.1%)	0.464
Tumor number			
1	23 (62.2%)	45 (57.7%)	0.800a
2	6	20	
3	7	9	
4	1	4	
Size (Diameter, cm)	4.0±3.0	4.6±3.1	0.333
Ascites	3 (8.1%)	5 (6.4%)	0.711
Alpha-fetoprotein (ng/mL)b	50.3 (3∼9688)	33.0 (3∼16057)	0.165c
Albumin (g/dL)	3.8±0.6	3.9±0.6	0.775
Bilirubin (mg/dL)	1.0±0.5	1.4±0.8	0.202
Prothrombin time (sec)	12.2±1.1	12.2±1.3	0.940
Creatinine (mg/dL)	1.2±1.1	1.2±0.8	0.849
AST (U/L)	72.1±56.8	88.7±128.8	0.456
ALT (U/L)	73.9±57.5	106.4±185.4	0.300
Alcoholism	1 (2.7%)	16 (20.5%)	0.026
Detectable serum HBsAg	14 (37.8%)	21(26.9%)	0.389
Detectable Hepatic HBV DNA but undetectable serum HBsAg	4(10.8%)	12(15.3%)	0.578

a Comparison between patients with tumor number = 1 and those with tumor number >1.

b Median (range).

c Mann-Whitney test.

### Virological analysis using noncancerous liver tissues adjacent to HCC

Both HCV-RNA and HBV-DNA were extracted from noncancerous liver tissue for virological analysis. Of all patients, 58.2% (67/115) had HCV RNA levels greater than the lower detection limit for quantitative assessment (1500 IU/g) and 23.47% (27/115) had HCV RNA levels >13×106 IU/g (the cutoff used for survival analysis; Table 3). Among those with HCV RNA >1500 IU/g, 68.1% (45/66) were genotype 1b. For those who had either occult or overt HBVCI, 25.4% (13/51) had HCV RNA levels >13×106 IU/g and 66% (26/39) were genotype 1b. For those who had neither overt nor occult HBVCI, 21.8% (14/64) had HCV RNA levels >13×106 IU/g and 64.1% (14/25) were genotype 1b. No significant difference was found for HCV RNA levels and genotype distribution between patients with and without HBVCI.

**Table 3:** Univariate and multivariate analysis of clinicopathologial and virological parameters for disease-free survival in HCV-associated HCC.

Parameter	No. of patients	Mean disease-free survival (95% CI)	HR (95% CI)	Adjusted HR (95% CI)
Age (years)				
≦47	11	91.7(62.5∼120.91)		
>47	104	42.58(34.14∼49.97)	3.28(1.024∼10.508)*	2.018(0.067∼6.599)
Gender				
Female	37	48.0 (34.5∼61.5)		
Male	78	51.8 (38.9∼64.6)	1.099 (0.631∼1.917)	
Cirrhosis				
No	38	56.7 (37.6∼75.8)		
Yes	77	45.3 (36.2∼54.4)	1.064 (0.615∼1.841)	
Alcoholism				
No	98	58.2 (46.1∼70.3)		
Yes	17	39.9 (24.6∼55.2)	1.393 (0.752∼2.581)	
Tumor characteristics				
Microvascular invasion				
No	76	53.4 (40.8∼66.0)		
Yes	38	45.2 (31.0∼59.3)	1.132 (0.654∼1.962)	
Edmondson's grading				
I-II	40	55.9 (38.4∼73.4)		
III-IV	75	46.2 (36.6∼55.8)	1.018 (0.779∼1.331)	
Encapsulation				
No	26	48.1 (31.1∼65.1)		
Yes	88	48.5 (37.3∼59.7)	1.238 (0.653∼2.349)	
Tumor number				
1	68	53.5 (39.9∼67.1)		
>1	47	40.5 (28.3∼52.6)	1.313 (0.782∼2.206)	
Largest tumor size (diameter, cm)				
≦3	42	71.2 (53.8∼88.6)		
>3	73	37.5 (28.5∼46.5)	2.167 (1.227∼3.827)*	2.079 (1.149∼3.761)*
Second branch macrovascular invasion				
No	101	50.0 (38.9∼61.1)		
Yes	14	53.8 (29.2∼78.4)	0.735 (0.311∼1.737)	
AFP (ng/mL)				
≦8	19	70.9 (54.9∼86.9)		
>8	96	46.2 (35.2∼57.3)	3.259 (1.298∼8.183)*	5.976 (2.007∼17.794)*
Ascites				
No	107	51.5 (41.0∼62.0)		
Yes	8	50.8 (30.4∼71.3)	0.370 (0.051∼2.673)	
Albumin (g/dL)				
≦4.0	68	34.0 (24.1∼44.0)	2.263(1.312∼3.904)*	2.539(1.399∼4.606)*
>4.0	47	68.4 (53.3∼83.5)		
Bilirubin (mg/dL)				
≦1.0	48	62.6 (45.5∼79.8)		
>1.0	67	40.8 (31.4∼50.3)	1.423 (0.833∼2.431)	
Prothrombin time (sec)				
≦12	67	58.3 (44.0∼72.6)		
>12	48	39.9 (28.5∼51.4)	1.081 (0.951∼1.229)	
Creatinine (mg/dL)				
≦1.2	89	47.9 (37.0∼58.8)		
>1.2	26	59.6 (42.1∼77.2)	0.751 (0.517∼1.091)	
AST (U/L)				
≦42	49	71.9 (55.2∼88.6)		
>42	66	34.4 (25.5∼43.4)	1.253 (1.088∼1.443)*	1.050 (0.870∼1.266)
ALT (U/L)				
≦50	49	67.5 (50.9∼84.1)		
>50	66	38.4 (28.7∼48.1)	1.071 (1.008∼1.137)*	1.086 (1.006∼1.172)*
AST/ALT				
≦0.89	55	56.27(45.70∼66.83)		
>0.89	60	40.7(26.95∼54.45)	2.072(1.23∼3.491)*	1.621(0.944∼2.784)
Fat metamorphosis				
No	76	46.68(34.55∼58.80)		
Yes	39	52.97(40.01∼65.94)	0.68(0.39∼1.18)	
Virological factors				
HCV-RNA (all patients)				
≦13×106 IU/g	88	43.1 (34.6∼51.6)		
>13×106 IU/g	27	66.4 (43.1∼89.7)	0.636 (0.322∼1.256)	
HCV genotype (all patients)				
Non-1	22	41.9 (28.3∼55.5)		
1	45	59.5 (41.5∼77.6)	0.776 (0.386∼1.561)	
HCV-RNA (non-HBVCI patients only)				
≦13×106 IU/g	50	36.9 (25.7∼48.1)		
>13×106 IU/g	14	54.0 (37.9∼70.1)	0.355 (0.108∼1.168)	
HCV genotype (non-HBVCI patients only)				
Non-1	14	28.6 (17.0∼40.3)		
1	25	53.3 (34.6∼72.0)	0.531 (0.202∼1.397)	
Intrahepatic HBV-DNA(By nested PCR detection)				
Negative	64	41.6 (31.1∼52.1)		
Positive	51	57.4 (42.5∼72.2)	0.771 (0.461∼1.291)	
Occult HBV superinfection				
No	99	56.9 (45.3∼68.5)		
Yes	16	29.2 (12.7∼41.8)	2.028 (1.069∼3.849)*	2.708 (1.317∼5.566)*
Overt HBV superinfection				
No	80	38.5 (29.6∼47.5)	2.062(1.14∼3.73)*	2.216(1.15∼4.269)*
Yes	35	70.4 (52.3∼88.6)		

* P<0.05.

HR, hazard ratio; CI, confidence interval.

Among the 35 patients with positive serum HBsAg (i.e. overt HBVCI), it was found that the HBV DNA level was lower than the detection limit of our quantitative test (120 IU/gram of liver tissue) in 3 patients. All the 3 patients were positive for hepatic HBV DNA on nested PCR assay. Of the 16 patients with occult HBVCI, 9 had HBV-DNA levels >120 IU/g, whereas in the remaining 7 patients, hepatic HBV DNA was positive only when nested PCR assays were performed. The intrahepatic HBV DNA levels varied greatly regardless of the status of serum HBsAg. For those with occult HBVCI, the intrahepatic HBV DNA levels ranged from undetectable to 3.4×105 IU/g; for those with overt HBVCI, the levels ranged from undetectable to 2.0×107 IU/g.

### Comparison between serum and tissue HCV-RNA and HBV DNA levels

As reported previously, tissue HCV-RNA levels were higher than the detection limit in 94 patients but were lower than the detection limit in 21 patients. Of the 94 patients, 82 (87.2%) had serum samples available for quantitative measurement, which were all found to be positive for HCV-RNA. Regression analysis showed a positive correlation (beta  = 0.012; 95% CI = 0.003 to 0.020; P = 0.007). Of the 21 patients with undetectable HCV-RNA in liver tissues, 17 (81.0%) had serum samples available for quantitative test. Of them, 5 were positive for HCV-RNA in the serum (125 to 2.8×105 IU/mL), whereas 12 were negative for HCV-RNA test. Interestingly, 10 of the 12 patients were positive for HBV-DNA in the liver tissues by PCR test, suggesting a suppression effect on HCV by HBV. Of these 10 patients, HBsAg was positive in 9 of them. Taken together, although 12 of our patients were negative for HCV-RNA in the tissue and serum samples, it was likely caused by viral interference in HBV+HCV coinfection. Therefore, in this study, we considered all anti-HCV positive patients as HCV-related HCC but with HBV superinfection as a variable for prognosis analysis.

In 51 patients who had positive tissue HBV-DNA by PCR assay (overt or occult HBV coinfection), 40 (78.4%) had serum samples available for quantitative analysis. Of them, 10 patients who had tissue HBV DNA <120 IU/g were also negative for serum HBV DNA. In the remaining 30 patients, the serum HBV-DNA was above detection limit. Regression analysis showed a positive correlation (beta  = 0.170; 95% CI = 0.044 to 0.297; P = 0.010).

### Clinicopathological and virological parameters associated with postoperative survivals in HCC

Cox proportional hazard model was used to examine the association between clinicopathological and virological factors, and disease-free survival after surgical resection of HCV-associated HCC (Table 3). Univariate analysis revealed that age >47 years old, tumor size >3 cm, alpha-fetoprotein >8 ng/mL, albumin ≤4 g/dL, AST >42 U/L, ALT >50 U/L, AST/ALT >0.89, presence of occult HBVCI, and absence of overt HBVCI were associated with a shorter disease-free survival. After adjusted for other confounding factors (Table 3), multivariate analysis revealed that tumor size >3 cm (*P* = 0.007), alpha-fetoprotein >8 ng/mL (*P* = 0.013), albumin ≤4 g/dL (*P* = 0.003), ALT >50 U/L (*P* = 0.022), presence of occult HBVCI (*P* = 0.027), and absence of overt HBVCI (*P* = 0.015) associated with a shorter disease-free survival.

**Figure 1:**
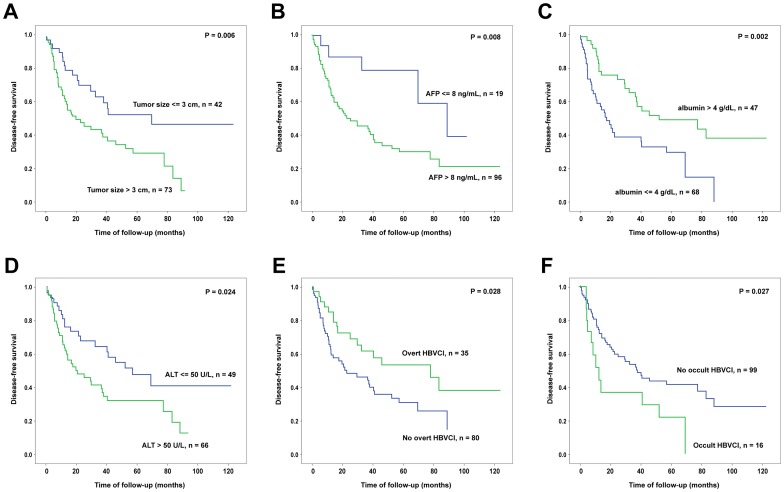
Kaplan-Meier analysis of the postoperative disease-free survival in HCV-associated HCC patients classified by each of the 6 independent predictors. Patients separated into two groups according to tumor size (A), alpha-fetoprotein (AFP) (B), albumin (C), ALT (D), overt HBVCI (E), and occult HBVCI (F) respectively were subjected for survival analysis. Green line, patients with assayed values greater than the cutoff (A to D) or patients with HBVCI (E and F); Blue line, patients with assayed values lower or equal to the cutoff (A to D) or patients with no HBVCI (E and F).

Kaplan-Meier survival analysis was thus performed to verify the predictive values of these 6 independent factors identified by multivariate analysis (Figure 1). It was found that in each of the factors, patients could be clearly separated into two groups with significant survival difference. Subsequently, we assigned a risk score to each of the patients by calculating the number of unfavorable independent predictors carried by each patient. The risk scores ranged from 0 to 6. Since the numbers of patients in some risk score subgroups were too small to achieve statistically significance, subgroups were combined to form three groups of patients with risk score ≤2, 3 to 4, and ≥5 (Figure 2A). The disease-free survivals were significantly different between any of the two groups. The disease-free survival was also compared among patients with occult HBVCI, overt HBVCI, and HCV infection only (Figure 2B). It was found that the disease-free survival was significantly different among the three groups (*P* = 0.015). Patients with overt HBVCI has a more favorable disease-free survival compared to those with HCV-infection only (*P* = 0.048) and with occult HBVCI (*P* = 0.002). No significant difference of disease-free survivals was found between those with only HCV infection and those with occult HBVCI (*P* = 0.182).

### Comparison of the clinical and virological factors among the various types of HBVCI in chronic hepatitis C patients with HCC

The clinicopathological factors were compared among patients with HCV infection only, occult HBVCI, and overt HBVCI (Table 4). ANOVA analysis and Post Hoc test revealed that patients with overt HBVCI had borderline significantly higher prevalence of fatty metamorphosis compared with those with HCV infection only (*P* = 0.043). Furthermore, among the three groups, patients with occult HBVCI had a significantly higher bilirubin level (*P* = 0.003). Otherwise, no significant difference was found among the three groups in terms of basic clinicopathological data.

**Table 4:** Comparison of basic clinical parameters between HCC patients classified by various types of HBV and HCV infections.

	HCV Ab (+)			
Classification	HCV infection alone	HCV infection with occult HBVCI	HCV infection with overt HCVCI	P
Case number	64	16	35	
Sex (male)	45 (71.4%)	12 (75%)	21 (60%)	0.47
Age (years)	64.5±9.44	63.0±11.8	59.4±12.8	0.073
Cirrhosis	43 (66.6%)	11 (68.75%)	23 (65.7%)	0.976
Fatty metamorphosis	17 (26.9%)	4 (25%)	18 (51.4%)	0.031a
Tumor number	1.6±0.9	1.6±0.7	1.8±0.9	0.299
Size (Diameter, cm)	4.2±3.0	5.9±3.5	4.2±2.7	0.113
Histology grade	2.61±0.60	2.87±0.8	2.8±0.71	0.1
Microvascular invasion	23 (36.5%)	6 (37.5%)	10 (27.5%)	0.523
Macrovascular invation	8 (12.7%)	1 (6.25%)	5 (14.2%)	0.717
Ascites	5 (7.9%)	1 (6.25%)	2 (5.7%)	0.921
Alpha-fetoprotein (ng/mL)	809 (2∼16057)	68.3 (0∼17141)	48.2 (0∼>100000)	0.9
Albumin (g/dL)	3.8±0.5	3.7±0.8	4.1±0.4	0.057
Bilirubin (mg/dL)	1.1±0.5	2.5±0.39	1.0±0.4	0.001b
Prothrombin time (sec)	12.1±1.2	11.9±1.0	12.4±1.6	0.442
Creatinine (mg/dL)	1.2±1.0	1.4±1.3	1.4±0.3	0.409
AST (U/L)	104.4±141.1 (24∼937)	67.6±60.5 (20∼201)	54.1±37.1 (13∼178)	0.088
ALT (U/L)	120.3±202.3 (18∼1462)	59.8±52.5 (17∼199)	70.3±61.7 (7∼286)	0.205
AST/ALT	1.06±0.71	1.21±0.47	0.93±0.40	0.292
Alcoholism	7 (11.1%)	5 (31.2%)	5 (14.2%)	0.124

a Sheffe's Post Hoc test revealed patients with HCV infection alone had significantly lower percentage of fat metamorphopsis than those with overt HBVCI (*P* = 0.043).

b Sheffe's Post Hoc test revealed patients with occult HBVCI had the highest bilirubin level (occult HBVCI vs. HCV infection alone, *P* = 0.003; occult HBVCI vs. overt HBVCI, *P* = 0.003).

**Figure 3:**
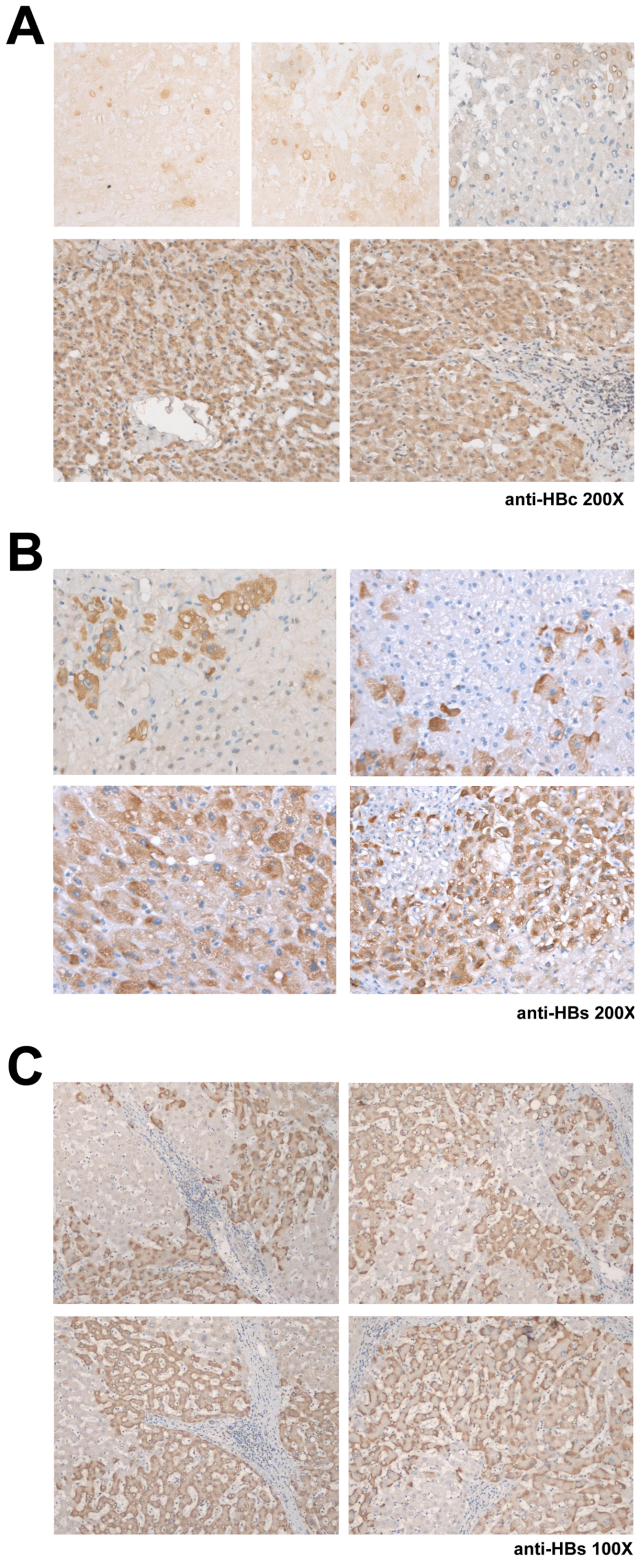
Representative immunohistochemistry results in HCV-associated HCC patients with occult HBVCI. (A) Tissue expression of HBcAg (200X). Upper panels from left to right: scattered distribution of nuclear staining (without counter stain), scattered distribution of nuclear membranous staining (without counter stain), and both nuclear and nuclear membranous staining (with counter stain). Lower panels, diffuse cytoplasmic staining. (B) Tissue expression of HBsAg (200X). Upper panels, scattered distribution of cytoplasmic staining. Lower panels, diffuse distribution of cytoplasmic staining. (C) Tissue expression of HBsAg (100X). Regional distribution of sub-membranous staining pattern.

Since in the HCV-associated HCC patients, those with occult HBVCI had significant shorter disease-free and overall survivals than those with over HBVCI, we compared the immunohistochemical and virological parameters between these two groups of HBVCI patients (Table 5). Immunohistochemical analysis revealed that more patients with overt HBVCI had positive tissue HBsAg staining in the noncancerous hepatic tissues than those with occult HBVCI, whereas there was a borderline difference of the percentage of patients with positive tissue HBcAg staining between the two groups (occult >overt, Table 5 and Figure 3A–C). In patients with occult HBVCI, despite undetectable serum HBsAg, tissue HBsAg was found in 5 patients presenting as a cytoplasmic or sub-membranous staining pattern in hepatocytes, with either diffuse or scattered distribution (Figure 3B and C). Additionally, in 3 of them, positive staining of HBcAg could be observed in the nuclei and/or cytoplasm of the hepatocytes and the distribution could be either scattered or diffuse (Figure 3A).

**Table 5:** Comparison of virological factors between occult and overt HBVCI patients with HCV-related HCC.

Virological factor	HBVCI		P
	Occult (n = 16)	Overt (n = 35)	
HBV-DNA (IU/g)	478 (0∼342826)a	2694 (0∼>2×107)a	0.041b
Tissue HBsAg expression	5(31.25%)	34(97.15%)	< 0.001
Tissue HBcAg expression	3(18.75%)	1(2.85%)	0.051
Precore G1896A	7 (43.8%)	3 (8.6%)	0.006
BCP mutations	4 (25.0%)	10 (28.6%)	0.999
Pre-S deletion	0	8 (22.9%)	0.045
HBV genotype C	3 (18.8%)	5 (14.3%)	0.694
HCV-RNA (× 106 IU/g)	3.2 (0∼94.7)a	0.4 (0∼49.2)a	0.215b
HCV genotype 1	8/10 (80%)	12/18 (66.7%)	0.669
HBV integration	5(31/2%)	14 (40%)	0.454
Survival			
Recurrence-free survival (months)	12.5 (5.42∼19.07)a	77.64 (26.7∼128.58)a	0.002c
Overall survival (months)	67.39±11.4d	115.145±4.6d	0.026c

a Median (range); b Mann-Whitney test; c Kaplan-Meier analysis; d Estimation is limited to the largest survival time if it is censored.

After Bonferroni correction for the alpha error, P<0.006 was considered statisitical significant.

Lower detection limit of intrahepatic HBV-DNA, 120 IU/g; Lower detection limit of intrahepatic HCV-RNA, 1500 IU/g.

**Figure 4:**
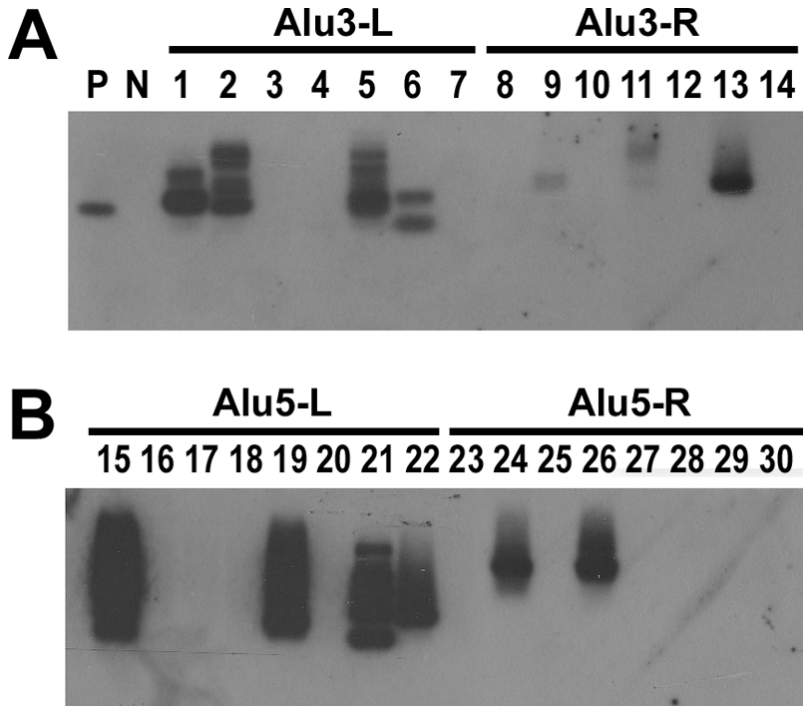
Southern blot analysis of Alu-PCR amplicons. Four sets of Alu-PCR were performed (See Table 1). (A and B) Alu3-L, Alu3-R, Alu5-L, and Alu5-R were the PCR products generated using primers Alu3/PCFo, Alu3/PCRo, Alu5/PCFo, and Alu5/PCRo respectively as the first stage PCR primers and Tag3/PCFi, Tag3/PCRi, Tag5/PCFi, and Tag5/PCRi respectively as the second stage PCR primers. The filter was hybridized with a digoxigenine-labeled HBV specific probe.

The genotypic characteristics of these 51 HBVCI patients were also assayed, including genotype, precore stop codon G1896A mutation, BCP A1762T/G1764A mutation, pre-S deletion mutation and sequence analysis of the whole S coding region (Table 5). Statistical analysis showed that the patients with occult HBVCI had a significantly higher rate of precore mutation (*P* = 0.006) but a borderline lower prevalence of pre-S deletion mutation (*P* = 0.045). HBV DNA levels in patients with occult HBVCI were lower than those with overt HBVCI. No difference was found in terms of genotype, BCP mutation rate and HBV integration rate assessed by Alu-PCR (Table 5 and Figure 4). Neither the occult nor the overt HBVCI patients carried amino acid mutation in or nearby the “a” determinant region of the S protein.

## Discussion

The most intriguing and unexpected finding of the current study was that occult rather than overt HBVCI led to shorter postoperative disease-free survival in patients with HCV-associated HCC. Overt HBVCI not only did not accelerate the recurrence of HCC but it suppressed its occurrence, resulting in longer disease-free survival. To decipher this puzzle, the clinicopathological and virological factors were compared between the two groups. As expected, patients with overt HBV infection indeed had a higher HBV DNA level and a higher rate of tissue viral antigen expression. However, several supposedly important oncogenic factors, such as HCV RNA level, HCV genotype, HBV genotype, HBV BCP mutation, and HBV integration rates, did not differ between the two groups. Despite these inconvenient findings, it was found that the patients with occult HBVCI had a higher bilirubin level and a higher prevalence of precore stop codon G1896A mutation.

Taiwan is a hyperendemic area of HBV infection with 15–20% of the general population chronically infected with HBV [28]. Majority of Taiwanese HBV carriers were infected with HBV perinatally before 3 years of age. Infection occurring after 3 years of age was less frequent and rarely developed into a chronic status [29], [30]. Hence, most cases enrolled in our study with dual HBV and HCV infection were likely chronic HBV carriers superinfected by HCV [31]. A reasonable assumption is that HBV serves as an initiator and HCV serves as a promoter for hepatocarcinogenesis in such patients. Therefore, a higher HCV RNA level should have associated with a higher HCC recurrence rate. In the present study, however, no significant difference was found for the intrahepatic HCV RNA level between these two HBVCI groups, despite the presence of significant survival difference. In fact, the intrahepatic HCV RNA level was not a predictor for postoperative survivals in any of our patient groups (Table 2). On the other hand, it was known that HBV genomic DNA can integrate into host chromosome, independently of the amount of free HBV DNA [32]. The integration potentially disrupts cellular genes and enhances chromosomal instability, leading to an increased risk of HCC development. However, in this study, the prevalence of HBV DNA integration was not significantly different between the occult and overt HBVCI patients.

It was not surprising that cases with overt HBVCI had higher intrahepatic HBV DNA levels than those with occult HBVCI. However, in 5 cases with occult HBVCI, high intrahepatic HBV DNA levels (up to 384286 IU/g, Table 3) were found, regardless of the negativity of serum HBsAg. The possibility that these patients harbored vaccine escape like mutations was excluded by sequence analysis of the whole S gene [22], [33]. It is therefore curious that in these patients, the presence of a high intrahepatic viral load of HBV did not result in a detectable amount of serum HBsAg. The fact that in these occult HBVCI patients, hepatic HBsAg and HBcAg were still detectable by immunohistochemistry suggested that there was a secretory defect for HBsAg (Figure 3). Intrahepatic retention of viral proteins could result in hepatocytopathy and subsequent necroinflammation, leading to an increased risk of postoperative recurrence. In the remaining 11 occult HBVCI patients, however, the intrahepatic HBV DNA was low and viral protein expression was undetectable. A possible explanation for the shorter disease-free survival in these patients is that they might be in a later stage of HCV and HBV co-infection. Presumably, in these patients, HCV and HBV might have taken turn to elicit numerous episodes of necroinflammation prior to suppression of HBV infection into the occult status. The poorer hepatic reserve in occult HBVCI patients (high bilirubin levels and higher AST/ALT ratios) might represent a liver suffered from more rounds of damage and thus carried a higher risk for postoperative HCC recurrence.

Whether a higher prevalence of the precore G1896A mutant in occult HBVCI patients had any effect on the increased risk of HCC recurrence remained to be determined. Reports regarding association between precore G1896A mutant and development of HBV-associated HCC were very conflicting so far [34], [35]. However, development of the precore G1896A mutation prevents the production of HBeAg, which provides a possible means to evade immune clearance and contributes to the persistence of occult HBVCI. Furthermore, precore G1896A mutation usually developed in patients suffering from repeated hepatitis flares during a long course of HBV infection. The increased prevalence of this mutation in occult HBVCI was consistence with the view that occult HBVCI was a later stage of HBV and HCV co-infection. In addition to the presence of occult HBVCI and absence of overt HBVCI, tumor size >3 cm, alpha-fetoprotein >8 ng/mL, albumin <4 g/dL, ALT >50 U/L, were also found to be independent predictors for the disease-free survival in the HCV-associated HCC patients. These results indicated that the degree of inflammation (ALT), hepatic functional reserve (albumin) and the tumor burden (AFP and tumor size) were three important factors associated with postoperative prognosis. Presumably, chronic hepatic necroinflammation with its subsequent generation of reactive oxygen species can induce chromosomal mutations and eventually lead to malignant transformation of proliferating hepatocytes. Likewise, poor liver function reserve, suggested by hypoalbuminemia, was found to be significantly associated with HCC occurrence in other studies [36]. By combining the 6 independent predictors, three groups with significantly different postoperative prognosis were distinguishable. Therefore, in an area hyperendemic for HBV infection, recognition of the status of HBVCI becomes mandatory for a better assessment of the postoperative prognosis. Finally, whether anti-HBV therapy should be given after operation for those who suffered from occult HBVCI becomes a viable question. Although microvascular invasion is usually recognized as important predictors in HCC patients undergone tumor resection [37], it was not present as the prognostic factor here. In our study, almost half (51/115) of the cases were co-infected with HBV. Necroinflammation elicited by host immune response to dual-infection with HBV and HCV [10], [11] may mask the predicting role of microvascular invasion in determining disease-free survival.

The major limitation of the current study is that the number of patients with HCV-associated HCC and occult HBVCI was relatively small. Nonetheless, this is the largest prospective study with 115 liver tissues obtained from the noncancerous parts of surgically removed HCV-associated HCCs worldwide so far (even in terms of the case number of occult HBVCI) [38]. Most studies trying to address the impacts of occult HBVCI on HCV-associated HCC were restricted by either inadequate HBV DNA sampling (from serum rather than liver) or small total cases number (cases number: 11∼85) [20], [38]. Furthermore, to our knowledge, there is still no study focusing on the role of occult HBVCI in postoperative prognosis of HCV-associated HCC patients. In Taiwan, HBsAg prevailed in 12% of anti-HCV-positive patients [39]. In our series, the prevalence of occult HBV CI was 20%. In view of this high prevalence, the roles of occult HBVCI in HCV-associated HCC cannot be overlooked in an area hyperendemic for HBV.

In conclusion, occult and overt HBVCIs were independent prognostic predictors for postoperative survival in HCV-associated HCC. In an area hyperendemic for HBV infection, recognition of the status of HBVCI is mandatory for a comprehensive assessment of the postoperative prognosis.
